# Whole blood RNA sequencing identifies transcriptional differences between primary sclerosing cholangitis and ulcerative colitis

**DOI:** 10.1016/j.jhepr.2023.100988

**Published:** 2023-12-19

**Authors:** Eike Matthias Wacker, Florian Uellendahl-Werth, Saptarshi Bej, Olaf Wolkenhauer, Mette Vesterhus, Wolfgang Lieb, Andre Franke, Tom Hemming Karlsen, Trine Folseraas, David Ellinghaus

**Affiliations:** 1 Institute of Clinical Molecular Biology, Christian-Albrechts-University of Kiel, Kiel, Germany; 2 Department of Systems Biology and Bioinformatics, University of Rostock, Rostock, Germany; 3 Indian Institute of Science Education and Research, Thiruvananthapuram, India; 4 Leibniz-Institute for Food Systems Biology at the Technical University Munich, Munich, Germany; 5 Stellenbosch Institute for Advanced Study (STIAS), Wallenberg Research Centre at Stellenbosch University, Stellenbosch, South Africa; 6 Norwegian PSC Research Center, Department of Transplantation Medicine, Division of Surgery, Inflammatory Diseases and Transplantation, Oslo University Hospital Rikshospitalet and University of Oslo, Oslo, Norway; 7 Department of Medicine, Haraldsplass Deaconess Hospital, Bergen, Norway; 8 Department of Clinical Science, University of Bergen, Bergen, Norway; 9 Institute of Epidemiology, Christian-Albrechts-University of Kiel, Kiel, Germany; 10 Research Institute for Internal Medicine, Division of Surgery, Inflammatory Diseases and Transplantation, Oslo University Hospital Rikshospitalet and University of Oslo, Oslo, Norway

**Keywords:** RNA-Seq, Gene Expression Profiling, Primary sclerosingCholangitis, UlcerativeColitis, Transcriptome, Whole blood, machine learning

## Abstract

**Background & Aims:**

Genetic and microbiome studies across patients with primary sclerosing cholangitis (PSC) and ulcerative colitis (UC) have indicated that UC in PSC is a separate disease entity to primary UC, but expression studies for PSC are lacking.

**Methods:**

We conducted whole blood RNA sequencing experiments for 495 patients with UC, 220 patients with PSC (including 177 with UC), and 320 healthy controls from Germany and Norway. Differential expression analyses, gene ontology and coexpression analyses and random forest machine learning were performed to identify genes, ontologies and transcriptional features that discriminate diagnoses.

**Results:**

The blood transcriptome in UC and PSC is dominated by neutrophil activation genes (*e.g. S100A12*). In UC, but not in PSC (neither PSC alone nor patients with an additional diagnosis of UC [PSC/UC]), ribosomal, mitochondrial, and energy metabolism genes are upregulated in conjunction with antibody transcript expression (*MZB1*, *IGJ*). In PSC, there is an increase in modules related to apoptosis and expression of genes of interferon-I-related ontologies. Random forest analysis could poorly discriminate PSC alone from PSC/UC (AUROC 0.56), but could discriminate PSC, UC, and controls with high accuracy (AUROC UC *vs.* controls 0.95, PSC *vs.* controls 0.88, UC *vs.* PSC 0.986). The main coexpression modules relevant for distinguishing PSC, UC, and controls are enriched in neutrophil degranulation and antibody production genes.

**Conclusions:**

Supported by machine learning results, PSC and UC appear to be separate entities on a molecular level, while PSC/UC and PSC are indistinguishable.

**Impact and implications:**

Clinical and genetic studies suggest that the colitis-like symptoms in primary sclerosing cholangitis (PSC) represent a different disease entity from primary ulcerative colitis (UC). The present study supports this assumption with transcriptomic data from whole blood and describes notable differences in gene expression between primary UC and PSC, providing insights into the still unclear pathophysiology of both diseases. These findings are of interest to scientists seeking to decipher the molecular pathophysiology of both diseases and provide evidence that a redefinition of the PSC-UC phenotype should be considered. The study practically supports future molecular research by providing a large transcriptomic whole blood reference cohort.

## Highlights

• Peripheral blood transcriptomes of UC are characterized by neutrophil activation, ribosomes, mitochondria, energy metabolism and antibodies.
• Patients with PSC exhibit higher expression of apoptosis and interferon-I-related coexpression modules.
• PSC and UC blood transcriptomes can be distinguished with machine learning.
• PSC with and without a concurrent UC diagnosis could not be distinguished.

## Graphical abstract

**Figure:**
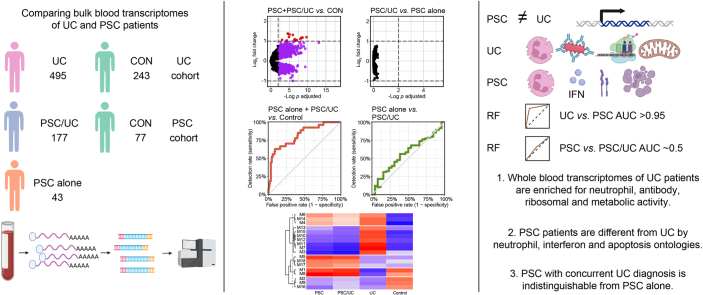

## Introduction

Ulcerative colitis (UC) is a chronic remitting and recurrent inflammatory disease of the rectum and colon and one of the two main types of inflammatory bowel disease (IBD). UC is thought to be the result of an inappropriate and persistent inflammatory response to commensal microbes in a genetically susceptible host.^1^ Nearly 50% of patients with IBD develop extraintestinal manifestations during their lifetime,^2^ sometimes including primary sclerosing cholangitis (PSC), a rare chronic liver disease characterized by inflammatory destruction of the intrahepatic and/or extrahepatic bile ducts and progressive liver disease.^3^ The prevalence of PSC is approximately 10 per 100,000,^4^ and 60-80% of patients with PSC also have IBD^5^ (referred to as PSC with concurrent IBD, although IBD is usually classified as ulcerative colitis so the condition is typically called PSC/UC). On the other hand, the prevalence of PSC in IBD varies between 0.5%^6^ and 8.1%.^7^ The clinical presentation of PSC/UC is different from primary UC,^3^ which suggests that they have different aetiologies.

Both PSC and UC have in common that their pathogenesis is largely unclear. The timing of disease onset and the expression of the IBD phenotype in PSC vary, with a general trend towards IBD preceding PSC.^8^ Genome-wide association studies for IBD and PSC have shown that UC and PSC share a substantial genetic component,^6^^,^^9^ although it is difficult to describe exactly what the common component is and how PSC/UC and UC differ at the molecular level. No coarse-grained taxonomic or functional differences were found between patients with PSC with and without IBD when changes in stool microbiome composition were examined.^10^

In this study, we present the transcriptomic differences between UC and PSC in case-control and cross-disease settings and demonstrate the utility of random forest machine learning models in identifying differentially expressed genes and predicting diagnosis from whole blood RNA sequencing samples. Using simultaneous RNA sequencing experiments on the same sequencers with the same protocols (see Methods) from whole blood samples of 495 patients with UC and 243 healthy controls from Germany and 220 patients with PSC (177 with a concurrent UC diagnosis) and 77 healthy controls from Norway (Table S1), we aimed to (i) elucidate differences and commonalities between UC, PSC alone and PSC/UC on the transcriptional level, (ii) establish transcriptome-based classifiers for UC, PSC alone and PSC/UC, and (iii) describe transcriptional characteristics of these diseases at the level of genes, pathways, cell types and coexpression modules. Disease-associated genes were identified using differential expression methods between cases and controls, random forest modelling, and coexpression analysis. To describe the blood transcriptome effects of a specific disease, it is necessary not only to study individual genes, but also to include information on the cell type in which a gene is expressed.^11^^,^^12^ For this purpose, we combined cell type-specific reference data^13^ with software for modular coexpression analyses^14^ and software for weighted correlation network analysis for coexpression analysis (WGCNA^15^) into a single methodological framework to improve the comparability of independent RNA sequencing (RNA-seq) datasets, in addition to software protocols for differential expression analysis^16^ and gene set-enrichment analysis (GSEA^17^).

Transcriptional biomarkers in blood and mucosa have already been proposed for UC and partially for PSC in a cross-disease analysis,^12^^,^[18], [19], [20], [21] and transcriptional models using machine learning techniques including penalized logistic regression have been developed to predict disease progression and treatment response in UC.^11^^,^^22^^,^^23^ However, these expression biomarkers and prediction models have not been independently replicated so far. Here, we show that our PSC and UC case-control and cross-disease modelling framework using random forest, a technique that has been shown to be powerful and robust in transcriptomic data,^24^ is applicable and replicable to multiple z-score transformed RNA-seq datasets and also valid across multiple independent UC RNA-seq datasets (no RNA-seq studies for PSC published to date to provide this proof of principle for PSC) from previously published RNA-seq studies from the US, Poland, Spain and Brazil,^12^^,^^19^^,^^21^ provided each cohort has its own sufficiently large control population for normalization purposes.

## Patients and methods

### Study participants

Written informed consent was obtained from all study participants and the institutional ethical review committees of the participating centers approved all protocols. The expression of genes was studied in peripheral whole blood samples from two case-control cohorts of patients using Lexogen/Illumina RNA-seq protocols as described in Uellendahl-Werth *et al.*^25^ The first cohort comprises 495 patients with UC and 243 healthy controls from Germany, the second cohort comprises 220 patients with PSC, of whom 177 were diagnosed with a comorbid UC (PSC/UC), and 77 healthy controls from Norway (Table S1). Patients and controls from Norway were provided by the Biobank of the Norwegian PSC Research Center, Oslo. For both cohorts, inclusion criteria were the respective, unambiguous diagnoses, an age of onset of at least 13 years and an age at sampling of 18 years. Treatments and duration of disease were not considered for inclusion. We refer to the disease itself as PSC, while PSC alone means the patients without a diagnosed concurrent UC and PSC/UC refers to patients with PSC and a concurrent UC diagnosis.

### Sample processing, sequencing

Total RNA was extracted with the QIAGEN (Hilden, Germany) PAXgene Blood miRNA Kit (Cat No./ID: 763134). All RNA isolates had an RNA integrity number value greater than 6 (first quartile 7.2, median 7.7, third quartile 8.0). The RNA isolates were stored at -80° Celsius until library preparation. Libraries were prepared with the Lexogen (Vienna, Austria) QuantSeq 3' mRNA-Seq Library Prep Kit and the Lexogen Globin Block Module for QuantSeq (Homo sapiens, Cat. No. 070.96). Globin Blocking reduces the proportion of haemoglobin transcript amplifications in whole blood samples directly during library preparation and enhances the signal of all other, more informative transcripts. Libraries for Illumina (San Diego, CA, USA) sequencing were prepared using 50 ng of RNA and 15 PCR cycles. Single reads of a length of 50 base pairs were sequenced on an Illumina HiSeq2500.

### Read data processing (nf-core/rnaseq)

Fastq-files were processed and mapped with the nf-core-rna-seq pipeline,^26^ (https://doi.org/10.5281/zenodo.2610144, version 1.3) to the GRCh37 genome. Genes were filtered by expression of at least 10 counts in 10% of the samples and haemoglobin transcripts were removed. Further analysis was carried out in R (v. 4.2.3^27^).

### Count data transformation (DESeq2 variance-stabilized, log-scale z-scores)

The R package DESeq2 Version 1.38.3^16^ was used to perform variance stabilizing transformation of the expression data using log-scale z-score stabilization.^24^ To account for library size, weakly expressed genes with high variances and outlier samples inflating variance, we required genes to have at least 10 reads in at least 10% of the samples in each cohort separately, used the variance-stabilized data (which is already on log-scale) provided by DESeq2 and centered the data on the mean of the controls of the respective cohort, scaling by variance of the control samples with outliers removed (>3 SD).

### Differential expression analysis

The R package DESeq2 Version 1.38.3^16^ was used for differential gene expression. The DESeq model accounted for potential sequencing plate batch effects and used the diagnosis or control state as outcome (design formula ∼Diagnose + PlateNr) and used the Wald-test. *P* values were Benjamini & Hochberg adjusted. We considered genes with an absolute log2-fold change of more than 1 and a Benjamini & Hochberg adjusted *p* value less than 0.01 significant.

### Machine learning classification models (random forest)

Machine learning was applied to predict the disease state based on expression data. It was performed with the glmnet^28^ and caret^29^ package and the rangeR^30^ implementation of the random forest algorithm. Random forest has proven effective in a wide range of transcriptomics settings,^24^ often better than logistic regression and the k-nearest neighbours method.

To apply a tuned ML model in two cohorts, the training and testing data were filtered to only contain the intersect of genes in both cohorts before training.

Binary classification predictions were carried out by equally splitting the dataset into training and testing datasets. The model was trained with 5-fold cross-validation on the training data with “average Gini impurity increase” importance^30^ (only random forest) to determine disease outcome based on all present expressions of genes. Best parameters for random forest were chosen based on a grid with mtry (1 – 20 and min_n (2 – 10) and for logistic regression with a l_1_ penalty of lambda (0 – 100). The model was then tested with the testing dataset and an optimal cut-off value was determined based on the classification probabilities. As the datasets were not highly imbalanced and to keep the results comparable to other studies the model performances were measured as AUROC. ROC curves were calculated and plotted with the R package pROC^31^ Version 1.18.0.

### Coexpression modelling (CEMiTool)

Weighted and signed coexpression networks were created with the R package CEMiTool Version 1.22.0^14^. CEMiTool internally calls WGCNA^15^ and automatically optimizes the soft-thresholding parameter β to a value of 8 (phi = 0.793, R^2^ = 0.805, dissimilarity threshold = 0.8, correlation method Pearson, minimum number of genes per module = 30). The tool also performed gene set-enrichment analysis (GSEA) and overrepresentation analysis on gene modules. Comparison of module activation between traits was calculated as normalized enrichment score (NES), as implemented in fgsea.^32^ Gene prefiltering was deactivated to enable a module assignment for every gene. The variable network_type was set to "signed" to receive modules with consistent sign of the log2-fold change. To enable the enrichment of the larger modules, the parameter gsea_max_size was increased to 10,000.

### Gene ontology set-enrichment analysis

TopGO^33^ is a GSEA R library, which includes various gene enrichment statistical methods against the gene ontology database “biological process" of the GO.db package. For enrichment of differential expression significant genes, we used the Fisher test and as background an expression-matched (baseMean) gene set, to reduce bias due to higher statistical power of strongly expressed genes. For enrichment of the most important genes on random forest, we used the top 50% cumulated importance genes as input and weighted those by feature importance, using the tie-tolerant variant of the Kolmogorov-Smirnov test (“ks.ties” in TopGO). Multiple testing correction was performed with the Benjamini-Hochberg (false discovery rate [FDR]) method. The *p* value threshold was 0.05 after FDR-correction for all gene ontology analyses.

### Overrepresentation analysis

For visualizing if genes from sets of differentially expressed genes or sets of most important genes in a random forest model are more often from a specific coexpression module than by chance, we simply calculated the overrepresentation factor as a ratio of two fractions:

$$r=\frac{\frac{n_{S\cap M}}{n_{S}}}{\frac{n_{M}}{n_{total}}}$$

where $n_{S\cap M}$ is the number of genes in the intersection of coexpression module and the prioritized gene set, $n_{S}$ is the number of genes in the gene set and $n_{M}$ is the number of genes in the coexpression module.

## Results

### Differential gene expression analysis and GSEA

**Fig. 1:**
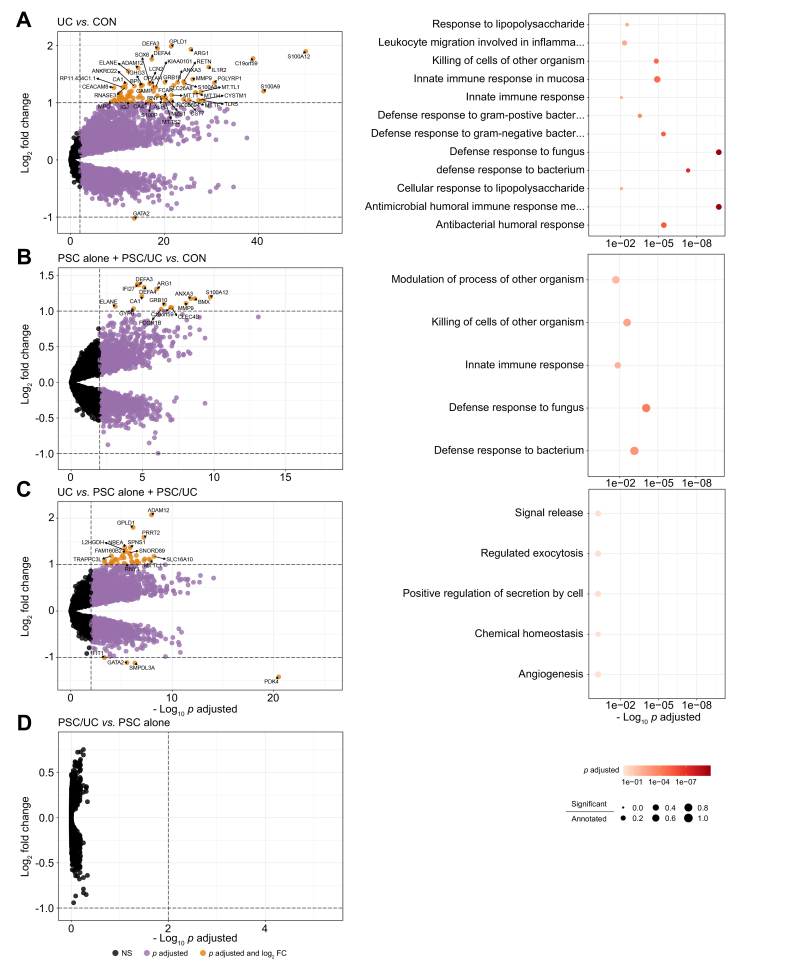
Results of differential expression analysis and gene ontology set-enrichment analysis. Left plots showed differentially expressed genes between (A) patients with UC *vs.* healthy controls (UC *vs.* CON), (B) patients with PSC alone or PSC/UC *vs.* healthy controls (PSC alone + PSC/UC *vs.* CON), (C) patients with UC *vs.* patients with PSC (UC *vs.* PSC alone + PSC/UC) and (D) patients with PSC/UC *vs.* patients with PSC alone (PSC/UC *vs.* PSC). Extensive dysregulation of transcripts was associated with innate immunity and is characteristic of UC and PSC compared to healthy controls resulting in significant enrichments at the gene ontology term level. Differences between UC and PSC seen at transcript level were not significantly enriched at gene ontology term level. There were no significantly dysregulated transcripts between patients with PSC without or with concurrent UC. Volcano plots: X-axis shows –log_10_ of the false discovery rate-adjusted Wald-test *p* values. Y-axis shows log_2_-fold change. Genes were considered significant with *p*-adjusted <0.01 and absolute log2-fold change >1. Biological function gene ontology term enrichment (Fisher test, Benjamini-Hochberg correction, *p*-adjusted <0.05) was based on significantly differentially expressed genes (red coloured genes from left volcano plots). CON, healthy controls; PSC, primary sclerosing cholangitis; UC, ulcerative colitis.

After sequencing, data pre-processing and quality control (see Methods), 11,377 genes were found to be expressed in peripheral blood of patients and controls. After data normalization, differential expression analysis (see Methods) was performed for patients with UC against controls (UC *vs.* CON), patients with PSC against controls (PSC alone + PSC/UC *vs.* CON), patients with UC against patients with PSC (UC *vs.* PSC alone + PSC/UC), and patients with PSC and concurrent UC against patients with PSC alone (PSC/UC *vs.* PSC alone) (Tables S2-5); 74, 15, 43 and 0 genes, respectively, showed significant differential expression (Benjamini & Hochberg *p*_FDR_ <0.01 and |log_2_-fold change| >1; Fig. 1). These gene lists were then analysed via GSEA for enrichment in gene ontology in the “Biological Pathways” database (Fig. 1, Tables S6-8, see Methods). For following comparisons of patients with PSC to controls or patients with UC, if not specified otherwise, we decided to pool those with PSC/UC and PSC alone, because we did not observe any differences between these groups that would justify a separate analysis. The overlap coefficient of differentially expressed genes (*p*-adjusted <0.01) between PSC alone *vs.* CON and PSC/UC *vs.* CON was 0.75.

Seventy-four differentially expressed genes, with the strongest associations for *S100A12*, *S100A9* and *C19orf59*, and gene ontology enrichment for patients with UC *vs.* controls suggested a strong innate immune response with terms associated with multiple areas of the immune response, including “defense response to bacterium/fungus”, “innate immune response” and “antibacterial humoral response” (Fig. 1A, Table S6). For PSC alone + PSC/UC *vs.* CON, there were 15 differentially expressed genes (*FCGR1B, S100A12, CLEC4D, IFI27, ELANE, C19orf59, MMP9, ANXA3, GYPB, ARG1, GRB10, DEFA4, DEFA3, CA1, BMX*) which were enriched for immune processes, with the strongest term being “defense response to gram-negative bacterium/fungus” (Fig. 1B). DEGs between UC and PSC alone + PSC/UC (Fig. 1C) were mostly upregulated in UC *vs.* CON and slightly downregulated in PSC *vs.* CON, except for *PDK4, SMPDL3A, GATA2, IFIT1*. No significant ontologies were obtained for UC *vs.* PSC alone + PSC/UC. No significant differences were found between PSC/UC and PSC alone (Fig. 1D).

### WGCNA and modular coexpression analysis

**Fig. 2:**
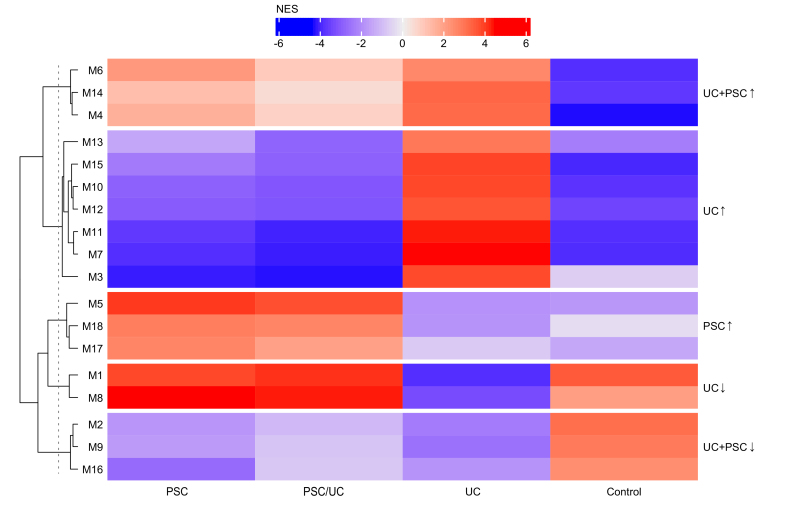
Weighted gene correlation network analysis and differential activation analysis of modules identified 18 coexpression modules clustered into five module groups. NES for patients with PSC without UC (PSC alone) and with comorbid UC (PSC/UC) were very similar in terms of expression of coexpression modules. NES for UC were very different from PSC alone, PSC/UC and healthy controls. Two NES values were considered different, with differences >2. Additional notes and an interpretation for these 18 modules can be found in Table 1 and in the Discussion. NES, normalized enrichment score; PSC, primary sclerosing cholangitis; UC, ulcerative colitis.

To contextualize genes from differential expression analysis, we identified coexpressed gene modules via WGCNA^15^ (see Methods). To determine the differential activation of modules in PSC/UC, PSC alone, and UC, we conducted modular coexpression analysis using CEMItool^14^ to our normalized data (see Methods). In total, we observed 18 coexpression modules with 41 to 2,550 genes in each module (Table S9), and the modules were strongly differentially expressed in UC, PSC and controls (Fig. 2). CEMiTool calculates the normalized enrichment score (NES) for each sample group and module. This score indicates whether genes in the module are overexpressed on average in a sample group compared to the other sample groups. PSC alone and PSC/UC were very similar, as NES per module did not differ more than two points for any module, whereas UC and controls differed significantly from PSC alone (Fig. 2). GSEA was performed on all modules, with most modules characterized by ontology terms (Table S10). Based on hierarchical clustering of coexpression modules, we assigned the 18 modules to five module groups with similar NES in each module group (Fig. 2). To provide a complete overview of our expression data, these were visualized in an overview heatmap (Figs S1-2). We observed strong dysregulation of expression much more frequently in UC than in PSC (with or without UC), which was more control-like.

**Fig. 3:**
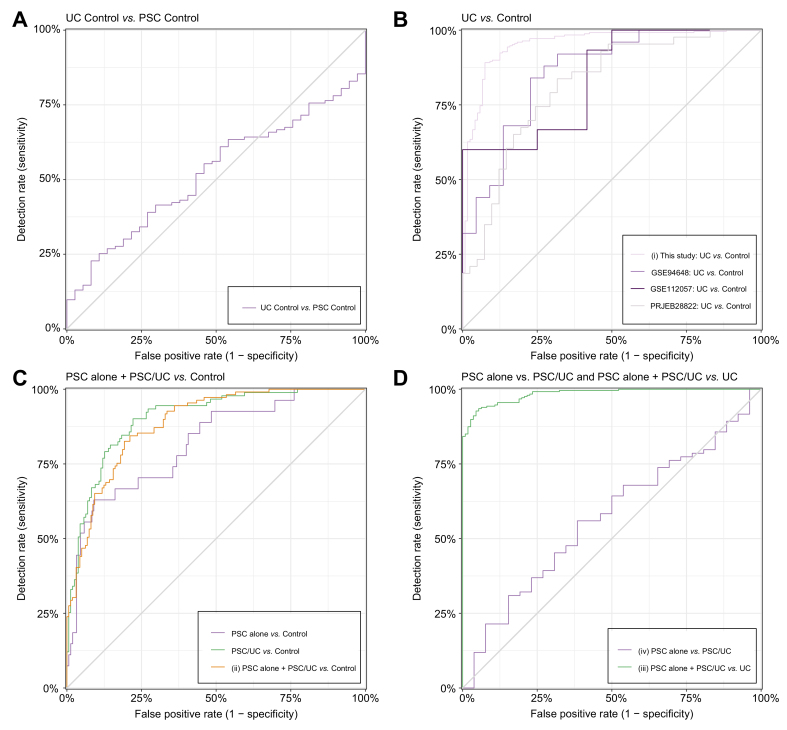
Random forest machine learning discriminates PSC, UC and controls with high accuracy, but poorly distinguishes PSC alone from PSC/UC. For sample sizes of validation and testing cohorts, see Table 2. (A) The healthy controls of UC and PSC cohorts cannot be distinguished by random forest. This serves as a negative control and shows that log-scale z-transformation of the data (see Methods) was successful. (B) UC can be distinguished from controls with high accuracy, AUROC 0.95 (95% CI 0.93-0.98). Our model was tested in three published transcriptomics datasets of patients with UC and controls (AUROC (95% CI): 0.86 (0.76-0.97, GSE94648^21^), 0.85 (0.71-0.99, GSE112057^12^), 0.81 (0.73-0.91, PRJEB28822^19^). (C) PSC alone + PSC/UC can be distinguished from controls with high accuracy (AUROC 0.88 (95% CI 0.84-0.92). A model using patients with PSC alone performs slightly worse due to the smaller sample size (AUROC 0.81 (95% CI 0.72-0.90). (D) Patients with PSC can be distinguished from those with primary UC with high accuracy, AUROC (95% CI) 0.986 (0.978-0.994), but PSC alone can be poorly distinguished from PSC/UC with AUROC (95% CI) 0.56 (0.44-0.68). 95% CI by DeLong’s method. PSC, primary sclerosing cholangitis; UC, ulcerative colitis.

To further annotate the gene expression modules, we examined the cell type-specific expression of each module using immune cell type-specific expression reference data as used in Cibersort^34^ (see Methods). We identified coexpression modules enriched in genes overexpressed by specific cell types (Table S11, Fig. S3B, Supplementary Methods), for example modules M4, M5, M8 and M14 were enriched for genes predominantly expressed in neutrophils. Further, we compared our modules to the blood transcriptomic axes described in Preininger *et al.*^35^ (Supplementary Methods, Table S12), which are nine conserved, large coexpression modules derived from seven independent expression datasets of blood. Four of our coexpression models matched one of the transcriptomic axes very specifically, suggesting they should be annotated in a similar way: M2 matched “B-cell activation”, M4 matched “cytokine receptor activity”, M6 matched “oxygen transporter activity”, and M17 matched “interferon-mediated signaling”. Summarizing these reference data annotations, we assigned modules, which were discussed in the paper, descriptive names for reference. These short names are not meant as exhaustive functional characterizations. Table 1 and Table S13 show a summary of our results from differential expression, gene ontology analysis, cell type-specific expression and blood transcriptomic axes for the 18 coexpression modules from Fig. 3.

**Table 1:** Annotation of 18 coexpression modules (from Fig. 2) by differential expression, gene ontology analysis, cell type-specific expression and blood transcriptomic axes.

Module	Number of genes	GO keywords	Expression cell type	Further observations	Module overrepresented in gene sets of DEGs and RF	Cluster	Short name
M6	573	Cell cycle, stress, signaling, metabolic, differentiation, ubiquitin, mitochond, wnt		GO terms "heme synthetic pathway", "erythrocyte differentiation"	(ii) PSC alone + PSC/UC *vs.* CON (DEG)	UC+PSC ↑	
M14	79	Neutrophil, B cell, defense, immune response, signaling	Eosinophils, Neutrophils	contains genes S100A9, S100A12, C19orf59	(i) UC *vs.* CON (DEG), (ii) PSC alone + PSC/UC *vs.* CON (DEG, RF) (iii) PSC alone + PSC/UC *vs.* UC (DEG)		Neutrophil-*S100A12*
M4	1,317	Neutrophil, T cell, platelet, cell death, immune response, signaling, metabolic, differentiation, RNA, mRNA, ER, transcript, interleukin, NF-κB	Monocytes, Neutrophils	GO term "neutrophil activation involved in immune response" p-adjusted < 10e-39			Neutrophil-cytokine-apoptosis I
M13	80	Platelet, chemotaxis, metabolic, differentiation, migration		GO term "regulation of megakaryocyte differentiation"	(iii) PSC alone + PSC/UC *vs.* UC (DEG)	UC ↑	
M15	72			contains mitochondrial genes and S100A8	(i) UC *vs.* CON (DEG, RF), (iii) PSC alone + PSC/UC *vs.* UC (DEG, RF)		Mitochondrial genes
M10	400	T cell, cell cycle, stress, signaling, splicing, metabolic, differentiation, RNA, mRNA, rRNA, ncRNA, expression, ubiquitin, mitochond, ER, transcript, ribosom, interleukin, NF-kappaB, wnt	T cells CD4 memory activated	contains gene IGJ	(i) UC *vs.* CON (RF)		Translation-respiration I
M12	176		B cells naive	contains gene ADAM12	(i) UC *vs.* CON (DEG), (iii) PSC alone + PSC/UC *vs.* UC (DEG)		B cells-ADAM12
M11	209	Metabolic, RNA, mRNA, rRNA, ncRNA, expression, mitochond, ER, transcript, ribosom	T cells CD8, T cells CD4 naive, T cells CD4 memory activated, T cells follicular helper	contains gene MZB1	(i) UC *vs.* CON (RF), (iii) PSC alone + PSC/UC *vs.* UC (RF)		Translation-respiration II
M7	555			GO term "vesicle mediated transport"	(iii) PSC alone + PSC/UC *vs.* UC (DEG)		
M3	1,474	Metabolic, RNA, rRNA, ncRNA, ribosom	T cells CD4 naive				Ribosome biogenesis
M5	690	Neutrophil, macrophage, cell death, immune response, stress, signaling, metabolic, ubiquitin, transcript, interleukin, interferon, NF-kappaB	Monocytes, Eosinophils, Neutrophils	GO term "positive regulation of programmed cell death"	(ii) PSC alone + PSC/UC *vs.* CON (DEG)	PSC ↑	Neutrophil-cytokine-apoptosis II
M18	41	Ubiquitin	Mast cells resting, Eosinophils	GO term "iron ion homeostasis"			Ubiquitin-iron homeostasis
M17	55	Cell cycle, defense, immune response, signaling, metabolic, RNA, ubiquitin, mitochond, interferon	Tregs, Macrophages M1, Dendritic cells activated	large overlap with expression axis "interferon-mediated signaling"	(iii) PSC alone + PSC/UC *vs.* UC (DEG)		Interferon-response
M1	2,550	Cell cycle, signaling, splicing, metabolic, RNA, mRNA, ubiquitin, ER, golgi, transcript		GO term "DNA metabolic process", genes GATA2 and PDK4		UC ↓	Transcription-splicing
M8	493	Neutrophil, immune response, stress, signaling, RNA, mRNA, transcript	Eosinophils, Neutrophils	GO Terms "extrinsic apoptotic signaling pathway",	(iii) PSC alone + PSC/UC *vs.* UC (RF)		Neutrophil-protein transport-apoptosis
M2	2,063	B cell, signaling, splicing, metabolic, RNA, mRNA, tRNA, rRNA, ncRNA, expression, mitochondria, transcript, ribosome		related to expression axis "B cell activation"		UC+PSC ↓	RNA processing-nuclear export-B cell activation
M9	483			GO term "spliceosomal tri-snRNP complex assembly"			
M16	67	Defense, immune response	T cells CD8, T cells gamma delta, NK cells resting, NK cells activated	GO term "cellular defense response"			

Modules were evaluated by size, pre-selected keywords as an overview, specific expression in blood cell types (delta average expression z-score >0.5) and additional notable observations. Individual genes were highlighted if they were differentially regulated and relevant for functional annotation. It was also noted if a module was overrepresented in the DEGs or in the top 50% of cumulative Gini importance in a RF model (see Methods). A short name was given to modules who are important to the interpretation in the main text.

CON, healthy controls; DEGs, differentially expressed genes; GO, gene ontology; PSC, primary sclerosing cholangitis; RF, random forest; UC, ulcerative colitis.

### Transcriptome-based random forest classifiers for UC, PSC/UC, and PSC alone

Through *in silico* experiments we tested whether a random forest machine learning model (see Methods) can reliably identify patients with primary UC, PSC/UC and PSC alone, and which features (genes) contribute most to the model. Because patients with UC and PSC were from different cohorts and to allow for cross-experiment comparisons, we used a log-scale z-transformation of the data using the healthy controls from each cohort as a reference to account for and correct for batch effects (see Methods). The successful elimination of batch effects was visually confirmed using principal component analysis (Fig. S4). First, as a negative control, we trained a random forest model of the German controls *vs.* the Norwegian controls. The model did not perform better than chance (AUROC 0.52, 95% CI 0.42-0.62, Fig. 3A). Next, we used a random forest model with 50% of the data for training and optimized model parameters by 5-fold cross-validation, followed by using the other 50% for validation purposes (see Methods). We performed the random forest analysis in a similar manner to the differential expression analysis for the following comparisons: (i) UC *vs.* CON, (ii) PSC alone + PSC/UC *vs.* CON, (iii) UC *vs.* PSC alone + PSC/UC, (iv) PSC/UC *vs.* PSC alone. We observed high performance in models (i-ii) (AUROC 0.95 [95% CI 0.93-0.98] and AUROC 0.88 [95% CI 0.84-0.92 95% CI, respectively], Fig. 3B,C, Table 2 and Table S14). Model (iii) performed almost perfectly (AUROC 0.986 [95% CI 0.978-0.994], Fig. 3D). We expected model (iv) to be challenging since we observed no significant differentially expressed genes between PSC alone and PSC/UC (Fig. 3D). No random forest model performing better than chance could be obtained for model (iv) (AUROC 0.56 [95% CI 0.44-0.68], Fig. 3D). These findings with our transcriptomic data from whole blood samples thus support previous observations from clinical,^3^ genetic^6^^,^^9^ and stool microbiome^10^ observations that concurrent UC is not a distinct subtype of PSC, and that concurrent UC in PSC and primary UC are different diseases.

We further validated the UC *vs.* CON model in three independent published datasets generated with different experimental transcriptome techniques, comprising 15, 46, and 25 patients and 12, 49, and 20 controls, respectively (Table 2 and Table S15). One of the studies was performed with paired-end Illumina RNA sequencing (GSE112057^12^), the second with the Ion Proton platform (PRJEB28822^19^) and the third was performed on Affymetrix microarrays (GSE94648^21^), showing generalisability of our model across platforms, as our data was generated with single-end 3’-tag sequencing on Illumina machines. Only one replication cohort was available^20^ for our PSC case-control model, but the performance of our PSC-CON model was weak in this dataset (Table 2), maybe because of clinical differences between the cohorts regarding age and stage of disease. Data normalization based on healthy controls in each UC cohort was performed as in the primary analysis to demonstrate robustness of our approach, leading to generalisable classification models. We tested the final classification model (i) (UC *vs.* CON) directly on the normalized datasets. Performances in our independent testing data sets were significantly lower than in our validation set, as expected, but still high (AUROC (95% CI): 0.86 (0.76-0.97), 0.85 (0.71-0.99), 0.81 (0.73-0.91) Fig. 3B, Table 2 and Table S14), showing that our random forest approach is powerful and applicable across cohorts using the log-scale z-score transformation. For comparison, we also fitted a logistic regression model with l_1_-penalization to the UC *vs.* CON model and tested it on the three validation cohorts but observed less robust performance (Table 2 and Table S14).

**Table 2:** Performance of transcriptome-based classifiers of a random forest analysis in the UC and PSC case-control cohorts and in additional testing cohorts available for UC.

Model name/dataset	N in dataset (cases *vs.* controls)	N training	N validation	AUROC validation	N testing	AUROC testing
(i) UC *vs.* CON	738 (495+243)	335	335	0.95 (0.93-0.98)		
GSE94648^21^	27 (15+12)				27	0.86 (0.76-0.97)
GSE112057^12^	95 (46+49)				95	0.85 (0.71-0.99)
PRJEB28822^19^	45 (25+20)				45	0.82 (0.73-0.91)
(ii) PSC alone + PSC/UC *vs.* CON	297 (220+77)	149	148	0.88 (0.84-0.92)		
GSE119600^20^	92 (45+47)				92	0.55 (0.43-0.67)
(iii) UC *vs.* PSC alone + PSC/UC	715 (495+220)	324	323	0.986 (0.978-0.994)		
(iv) PSC alone *vs.* PSC/UC	220 (43+177)	110	110	0.56 (0.44-0.68)		
Negative control: UC controls *vs.* PSC control	320 (243+77)	160	160	0.52 (0.42-0.62)		

N stands for the size of the complete dataset and the two classes to be distinguished, e.g. patients with UC and healthy controls. N training is the number of samples used for training the model, N validation is the number of validation samples to calculate the AUROC with 95% CIs (by DeLong’s method) in the German and Norwegian data. Model (i) was further tested three times in independent UC case-control data sets (for details, see Table S15). N.a. means that there are no independent test datasets for PSC and controls so far.

CON, healthy controls; PSC, primary sclerosing cholangitis; UC, ulcerative colitis.

We also performed biological pathway GSEA on the gene set with the highest cumulative importance from training for each random forest model of Fig. 3, weighting genes by average Gini impurity decrease (Tables S16-S18; see Methods). We found that for (i) UC *vs.* CON, the term “cytoplasmic translation” is the only significant one after multiple testing correction (Tables S16 and S17). Not only known marker genes, such as *S100A12* and *S100A9,* but also genes encoding ribosomal components and mitochondrial genes (examples including *COX6B1*, *MT-RNR2*, *RPS9*) were among the top 10 most important genes (Table S19). In (ii) PSC alone + PSC/UC *vs.* CON, no significant associations were found, although immune system terms are suggestive. For (iii) UC *vs.* PSC alone + PSC/UC, “cytoplasmic translation” is the only significant term, as in (i) UC *vs.* CON (Table S18). There are consistent transcriptional changes related to ribosomal components useful for machine learning in (i) UC *vs.* CON which are not observed in (ii) PSC alone + PSC/UC *vs.* CON.

### Prioritization of coexpression modules

To prioritize the 18 coexpression modules from the WGCNA that are most informative for UC and PSC, we tested whether differentially expressed genes and highly important random forest genes were overrepresented in certain modules. We calculated an overrepresentation factor indicating how many genes of such a set were assigned to a module than expected by chance (Table 1, Fig. S5, see Methods). We observed that (i) UC *vs.* CON and (iii) UC *vs.* PSC alone + PSC/UC were similar in terms of the most overrepresented modules. For differentially expressed genes, modules M12 (“B cells-ADAM12”) and M15 (“mitochondrial genes"), and for random forest, modules M11 ("translation-respiration II”) and M15 were enriched in both comparisons. For (ii) PSC alone + PSC/UC *vs.* CON, module M14 (“neutrophil-S100A12”) seems to be particularly relevant from differentially expressed genes. For (iv), analogous to the previous results and the poor discrimination of PSC/UC and PSC only, there was no relevant enrichment.

First, to derive specific features of UC from these prioritization results, we focused further on modules M11, M12 and M15. These modules were characterized by genes predominantly expressed in T and B cells (Fig. S3B, Table S11) and genes encoding ribosomal and mitochondrial proteins. M11 (“translation-respiration I”) contains *MZB1*, which is involved in positive regulation of IgA expression^36^ and is significantly overexpressed in UC (Table 1). This gene is linked to module M10 ("translation-respiration I”), which is also disproportionally relevant for the (i) UC *vs.* CON random forest model and is overexpressed in (i, iii) UC compared to controls and PSC (Table 1, Fig. S5). M10 is enriched for genes expressed in T cells and contains *IGJ* (*JCHAIN*) which is significantly overexpressed. *MZB1* has been shown to promote *IGJ*-containing IgA secretion in the gut, which is critical for suppressing gut inflammation,^36^ but IgA is also expressed and functional in blood.^37^ Overall, we hypothesise that these modules are proxies for antibody production, which seems to be more active in UC compared to controls, PSC alone and PSC/UC. We looked up immunoglobulin genes in our data, which are strongly upregulated in UC but not PSC (Table S20), and found that IGHA1, IGHG1, IGHG3, IGLC2, IGLC3, IGJ and IgA receptor FCAR (CD89) are moderately to strongly and significantly overexpressed in (i) UC *vs.* CON but not in (ii) PSC+PSC/UC *vs.* CON, where only IGHG3 and FCAR are slightly overexpressed. Next, to derive specific features of UC and PSC from these prioritization results, we focused on M14 (“neutrophil-S100A12”), which is overrepresented among differentially expressed genes of (ii) PSC+PSC/UC *vs.* controls, although for random forest results, modules were almost equally important for correct classification. For (i) UC *vs.* CON, M14 was also relevant, so upregulation of M14 is probably a common feature of UC and PSC. Functionally, transcripts in M14 are positive regulators of inflammatory response and neutrophil degranulation and are abundant in neutrophils and eosinophils. M14 contains *S100A12, S100A9 and C19orf59,* overexpression of which has been frequently observed in the blood of patients with UC.^12^^,^[18], [19], [20], [21]

As we were interested in specific features of the PSC transcriptomes, we further investigated modules M5 ("neutrophil-cytokine-apoptosis”), M17 (“interferon-response”) and M18 (“ubiquitin-iron homeostasis”) because they were specifically upregulated in PSC but not in UC (Fig. 2). M17 is enriched for genes of the type I interferon-response (Table 1 and Table S10). It is also enriched for genes that are highly expressed in M1 macrophages and activated dendritic cells (Table 1, Fig. S3B). M18 is enriched for ubiquitin-related processes, iron metabolism and genes expressed in mast cells and eosinophils (Table 1). M5, mainly expressed in neutrophils, eosinophils and monocytes, contains ontologies such as “neutrophil mediated immunity” and “positive regulation of programmed cell death” (Table 1). UC and PSC share some strongly upregulated genes of this module, such as *ELANE, DEFA3 and DEFA4,* which according to gene ontology encode neutrophil proteins of the antimicrobial response (Tables S2,3,10,13). The cell death-related component of the module seems to be downregulated in UC. For example, M5 contains *G0S2*, a master apoptosis switch,^38^ which is upregulated in PSC (log2-fold change 0.74, *p*-adj = 2e-4, Table S2) but not UC (log2-fold change 0.18, n.s., Table S1).

## Discussion

The aim of this study was to determine transcriptomic features of PSC with and without concurrent UC (*i.e*., PSC/UC in the latter case), primary UC and healthy controls, and to highlight differences between these groups. First, we replicated the well-known upregulation of neutrophil-associated transcripts for patients with UC.^12^^,^^39^ For patients with PSC, we are only aware of one other publication providing transcriptomic data from peripheral blood^20^ although we were unable to replicate their differentially expressed gene sets or validate our findings in their expression data. For UC, we further observed an upregulation of genes of general cellular metabolism, like genes coding for ribosomal proteins, mitochondrial genes and genes of the oxidative phosphorylation process, which has partly been shown by others.^12^^,^^39^ We showed that although PSC and UC are both characterized by strong upregulation of genes like *S100A12, S100A9* and *C19orf59* in module M14 (“neutrophil-S100A12”) from WGCNA compared to controls, their transcriptomes are quite different. Random forest classification models can easily distinguish PSC and UC, but most coexpression modules are unevenly expressed in the two diagnoses. We discuss these differences between PSC and UC below. On the other hand, PSC alone and PSC/UC can neither be distinguished by random forest nor by differential expression (here we could not identify a single differentially expressed gene between these PSC subgroups), while the classification models for UC *vs.* CON, PSC alone + PSC/UC *vs.* CON and UC *vs.* PSC alone + PSC/UC allowed for a clear distinction. Thus, UC in patients with PSC does not appear to be a molecular comorbidity of two separate disease entities when whole blood bulk transcriptomic data is considered. These results are consistent with findings from clinical and genetic studies showing that primary UC and the occurrence of UC in patients with PSC are likely to be clinically and genetically distinct.^3^^,^^9^^,^^40^ The occurrence of UC in PSC may be, for example, a gradient of gastrointestinal symptoms that cannot be linked to a genetic^9^ or transcriptomic correlation in whole blood samples. Since we used a sensitive machine learning algorithm and many patients with PSC and did not even observe a result with suggestive significance, we conclude that bulk RNA-Seq is not an appropriate method to determine why some patients with PSC develop clinically observable UC symptoms and others do not. It might be worthwhile to investigate whether patients with PSC/celiac disease share the same transcriptomic phenotype as PSC/UC and PSC alone patients.

One of the main challenges of this study was that comparisons could be affected by batch effects and bias. We applied a z-transformation of the variance-stabilized data from DESeq2^16^ to our data, using the healthy controls of each data set as a reference for the mean and standard deviation of gene expression.^24^ First, we tested the successful removal of batch effects and effectiveness of this data transformation by principal component analysis. We then showed that random forest cannot classify the controls of cohorts better than chance. Finally, we showed that random forest models for UC trained on the cohort presented here performed well on three other independent testing cohorts of patients with UC that we transformed using the same method. Since the PSC cohort was run with the same library prep kit and Illumina sequencing machine, we believe that we minimised batch effects to the extent that our results are generalisable. Nevertheless, batch effects are unavoidable to some degree in RNA-seq experiments.

The blood transcriptomes of patients with UC and PSC can be distinguished from those of healthy controls, for example, by genes of the M14 “neutrophil-S100A12” module (Table 1, Fig. S5). Genes of this module are not only relevant for case-control classification, but are also important for predicting response to treatment in colitis.^11^ In the modules M4 “neutrophil-cytokine-apoptosis I” and M14 “neutrophil-S100A12”, we find many common genes highly upregulated in both diseases, and genes of pro-inflammatory neutrophil-specific expression are enriched in both modules. For UC, the occurrence of *S100A12* and neutrophil-related ontologies has been expected and shown by others,^12^^,^^19^^,^^21^^,^^39^ but has not yet been investigated for PSC. The modules are more upregulated in UC than in PSC. However, modules M5 “neutrophil-cytokine-apoptosis II” and M8 “neutrophil-protein transport-apoptosis” are also enriched for neutrophils, both in terms of ontologies and cell-specific expression. Overall, M5 and M8 are overexpressed in PSC compared to UC (Fig. 2), although M5 also contains a selection of strongly upregulated genes for UC (*FCGR1B*, *ECHDC3*, *MPO*, *ELANE*, *CEACAM8*, *BPI*, *DEFA4*, *DEFA3*). M5 and M8 have heterogeneous ontologies, in this case they are also significantly enriched for apoptosis terms "positive regulation of programmed cell death” and “extrinsic apoptotic signaling pathway”. M5 and M8 could reflect the apoptosis machinery of neutrophils. The role of apoptosis is unclear in PSC research,^3^ but a genome-wide association study has highlighted that apoptosis genes are differentially expressed in PSC,^41^ suggesting that it is relevant in PSC biology. The importance of apoptosis in regulating neutrophil activity is well known.^42^ In summary, neutrophils are activated in both diseases, but in PSC this could be balanced by increased apoptosis. However, it remains unclear whether this reflects the less severe colitis seen in patients with PSC.^3^^,^^40^

In addition to the modules mentioned above, other modules of the transcriptome are relevant, mainly for UC. It was shown previously that the strongest axis of variation was related to terms including neutrophils (upregulated) and general cellular biosynthesis or oxidative phosphorylation (upregulated) in patients with UC *vs.* controls.^12^^,^^39^ It is clear from the data presented here that cellular biosynthesis and metabolism are generally upregulated in UC. For example, genes for ribosomal proteins and the respiratory chain (starting with *(M)RPS, (M)RPL, SDH, COX* and *NDUF*) and mitochondrial genes are mostly upregulated in UC, and some have a high importance in the random forest classification model. This was not observed in PSC. Neutrophil activation depends on mitochondrial ATP release^43^ which was proposed as an explanation for the co-occurrence of neutrophil, respiratory and mitochondrial upregulation in the UC blood transcriptome study by Juzenas *et al.*^39^ The data from this study suggested that the upregulation of ribosomal and antibody transcripts in UC also needs to be elucidated. Most ribosomal protein genes are located in modules M10 “translation-respiration I” and M11 “translation-respiration II”, which were upregulated in UC. Modules M2 “RNA processing-nuclear export-B cell activation” and M3 “ribosome biogenesis” are both enriched in the term “ribosome biogenesis”, *i.e.* have more genes that support the production of ribosomes rather than expressing the actual components. Genes of the mitochondrial respiratory chain are mostly found in modules M2, M3 and M10, and mitochondrial tRNAs are an essential part of M15 “mitochondrial genes”, which is also highly upregulated in UC but not in PSC. At the same time, M10 and M11 are enriched for genes expressed in activated memory CD4 T cells and contain the significantly upregulated (in UC) genes for the J chain and essential IgA secretion protein *MZB1*.^36^ In the study by Preininger *et al.*,^35^ in which conserved coexpression modules were discovered in the blood of healthy adults, transcriptional axes (modules) #1 and #3 in blood are associated with translation, ribosome components, but also T-cell physiology and B-cell activation. These axes are reflected in our data in modules M2, M3, M9, M10 and M11 (Table S12). It seems reasonable that increased translational and metabolic functionalities are required for antibody production or expansion of cells with high translational capacities.

Another transcriptional difference between UC and PSC is the expression of *PDK4*, a usually tightly controlled kinase that inhibits the pyruvate dehydrogenase complex and thus the citric acid cycle, cellular respiration and oxidative phosphorylation.^44^ *PDK4* is upregulated in PSC but downregulated in UC and is the strongest differentially expressed gene between UC and PSC (Fig. 1C). This may point to a significant difference in energy metabolism in circulating immune cells from patients with UC and PSC. It was reported that neutrophil activation is regulated by mitochondrial ATP production.^43^ However, neutrophils appear to be activated in PSC as in UC, but oxidative phosphorylation machinery is not overexpressed in PSC, and *PDK4* is mainly expressed in monocytes in the blood.^45^^,^^46^ Although UC shows stronger and more distinct changes in transcriptomes, we observed M5 “neutrophil-cytokine-apoptosis II”, M17 “interferon-response” and M18 “ubiquitin-iron homeostasis” as coexpression modules which are upregulated in PSC but not UC and controls. The transcripts in these modules were associated with apoptosis, as described above, but also with interferon-signaling, probably linked by the strong upregulation of *IFI27* in PSC (log2-fold change 1.38, SD 0.27), which is known to affect apoptosis^47^ We consider these findings as new working hypotheses for disentangling UC and PSC/UC.

In the future, longitudinal samples of patients diagnosed with UC first who later developed PSC or were found to have PSC, could be of great value for the development of a predictive biomarker for PSC. The data presented here could serve as a reference dataset for a follow-up study with possibly fewer participants to help identify PSC at an early stage.

## Financial support

This work was funded by the nonprofit organization PSC Partners Seeking A Cure, grant project “A cross-disease map to identify concrete gene targets by characterizing expressional changes in PSC and UC”. The project received funding from the DFG (10.13039/501100001659Deutsche Forschungsgemeinschaft) grant no. EL 74 831/7-1 (project number 507145175). The study was further supported by The Norwegian PSC Research Center (http://ous-research.no/nopsc/), the German 10.13039/501100002347Federal Ministry of Education and Research (BMBF) within the framework of the e:Med Vernetzungsfonds funding concept (GB-XMAP; grant 01ZX1709A-C) and received infrastructure support from the 10.13039/501100001659Deutsche Forschungsgemeinschaft (DFG, German Research Foundation) Cluster of Excellence 2167 “Precision Medicine in Chronic Inflammation (PMI)” (EXC 2167-390884018), the DFG research unit "miTarget" (project number 426660215; INF (EL 831/5-1)) and the PopGen Biobank (Kiel, Germany; Ref Nr. 2017-006).

## Authors’ contributions

EMW, FUW and DE developed the study concept and design; DE supervised the study. TF, THK, MV, WL, AF and DE were involved in study subject recruitment, sample collection and assembling phenotypic data; EMW and FUW performed computational analyses. SB and OW helped preparing the data. EMW, FUW and DE wrote draft of the manuscript; All authors reviewed, edited and approved the final manuscript.

## Data availability statement

The expression data generated in this publication is accessible from Gene Expression Omnibus (https://www.ncbi.nlm.nih.gov/geo/) under the accession number GSE177044. All scripts used for analysis of the data are available via Github: https://github.com/ikmb/ucpsc-rnaseq.

## Conflict of interest

The authors declare no competing financial interests.

Please refer to the accompanying ICMJE disclosure forms for further details.
