# Supplementary material for: Whole blood RNA sequencing identifies transcriptional differences between primary sclerosing cholangitis and ulcerative colitis
Source: JHEP Rep. 2023 Dec 19;6(2):100988. doi: 10.1016/j.jhepr.2023.100988 (PMC10832281; doi:10.1016/j.jhepr.2023.100988)
Supplement: Multimedia component 1 [file mmc1.pdf]

Whole blood RNA sequencing identifies transcriptional differences between primary sclerosing cholangitis and ulcerative colitis

Eike Matthias Wacker, Florian Uellendahl-Werth, Saptarshi Bej, Olaf Wolkenhauer, Mette Vesterhus, Wolfgang Lieb, Andre Franke, Tom Hemming Karlsen, Trine Folseraas, David Ellinghaus

Table of contents

Supplementary Figures 2

Supplementary Methods 8

Supplementary Results 9

Supplementary References 10

Supplementary Figures

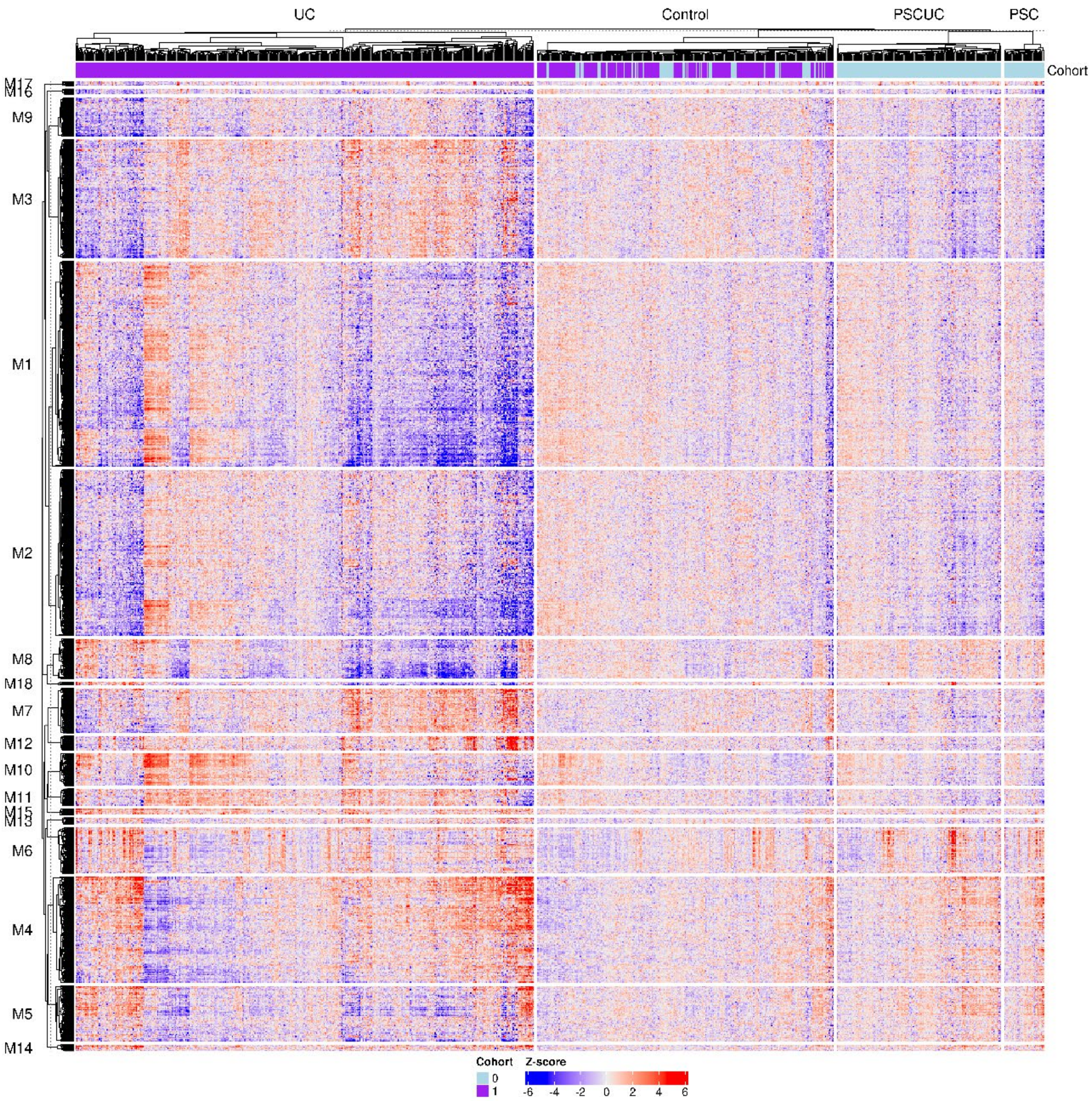

Fig. S1: Heatmap of gene expression z-scores (see Methods) by coexpression module, diagnosis and cohort.

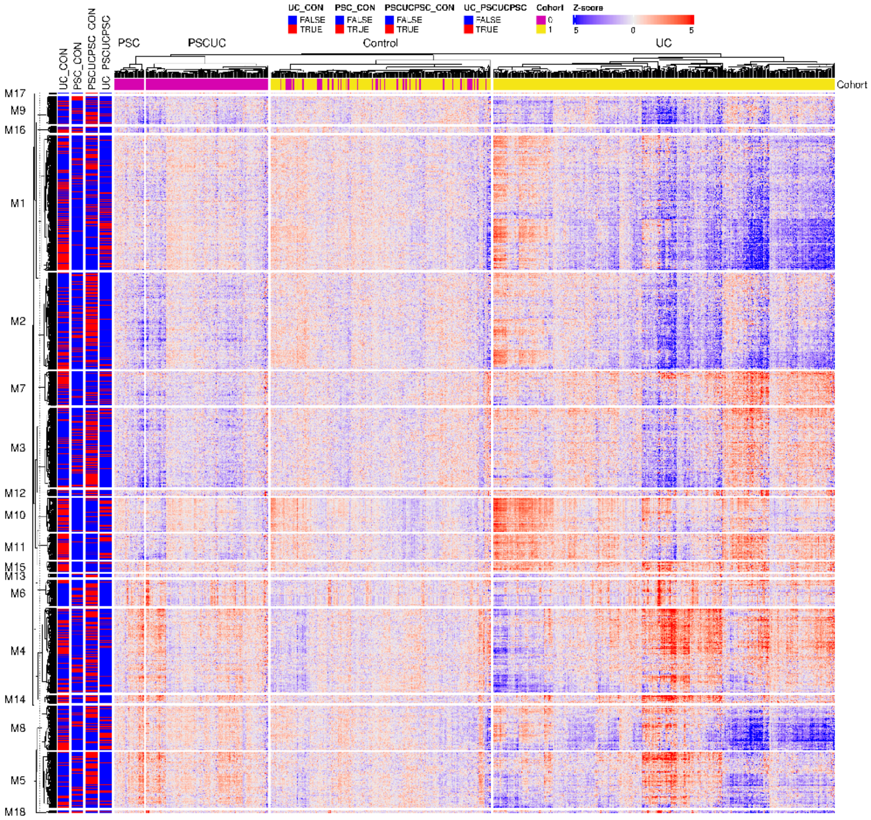

Fig. S2: Heatmap of gene expression z-scores (see Methods) filtered by genes that are in at least one of the top 50% importance gene sets for one of the random forest models. Y-axis is sorted by coexpression modules, x-axis by diagnosis and cohort.

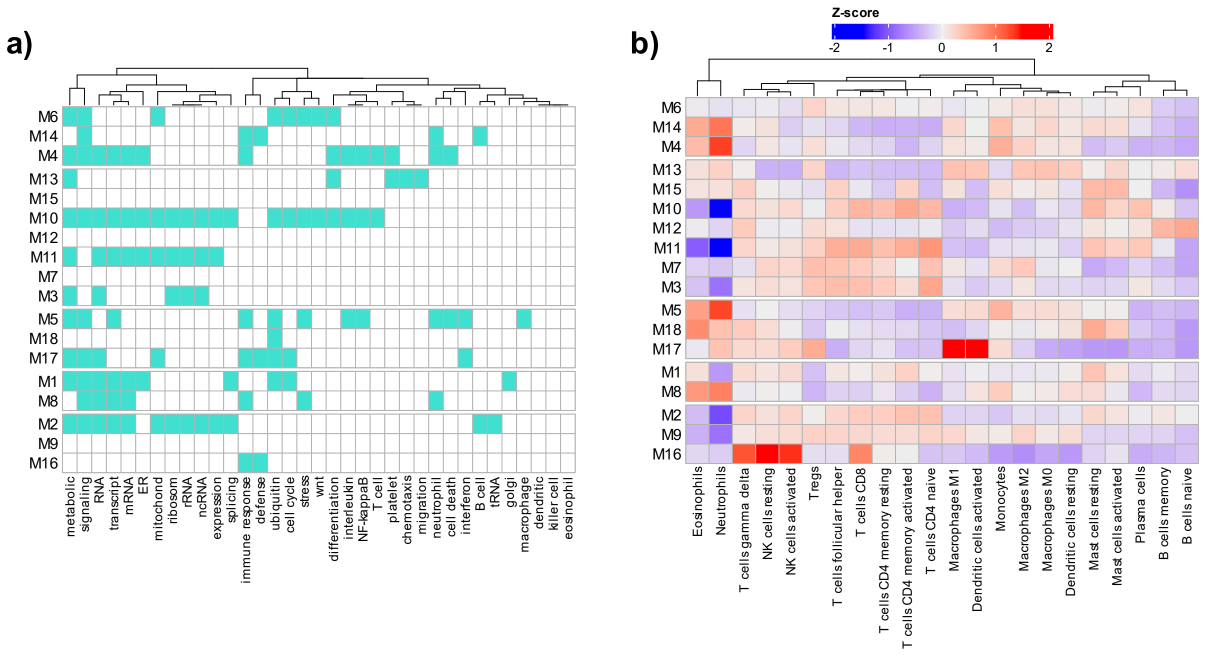

Fig. S3: a) Gene ontology enrichment for gene lists in co-expression modules give many results. For an overview, we selected some keywords and checked if each keyword occurs in the gene ontology enrichment result (Supplementary Table S10) of a coexpression module. b) We used the LM22 expression data to visualize if a gene is preferably expressed in a specific cell type (Supplementary Methods). The LM22 expression data was originally used to calculate the cell deconvolution signature matrix in Newman *et al.* 2015[1].

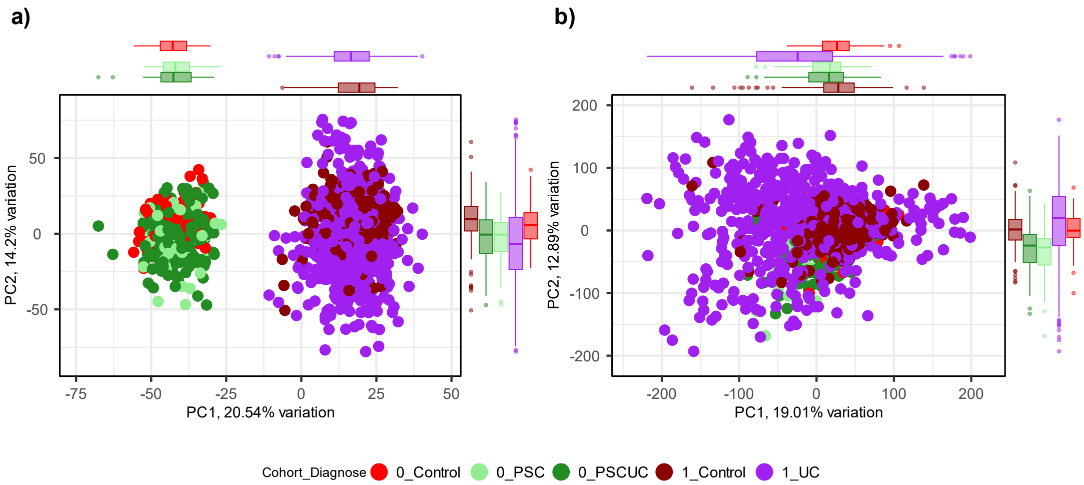

Fig. S4: Principal Component Analysis prior transformation indicates technical batch effects. a) PCA plots before and b) after z-score transformation of merged data sets of UC and PSC cohort. To merge our two cohorts and identify possible technical batch effects we applied principal component analysis (PCA). These effects were visible in PC1 that strongly differentiated between UC and PSC Cohort in Plot a). As both cohorts contained enough healthy controls we applied z-score transformation based on healthy controls, meaning we subtracted the outlier filtered (3 standard deviations) mean of controls from any samples for each log-scaled data set, divided by the standard deviation of the controls and repeated PCA. This was successful because both cohorts converge in b).

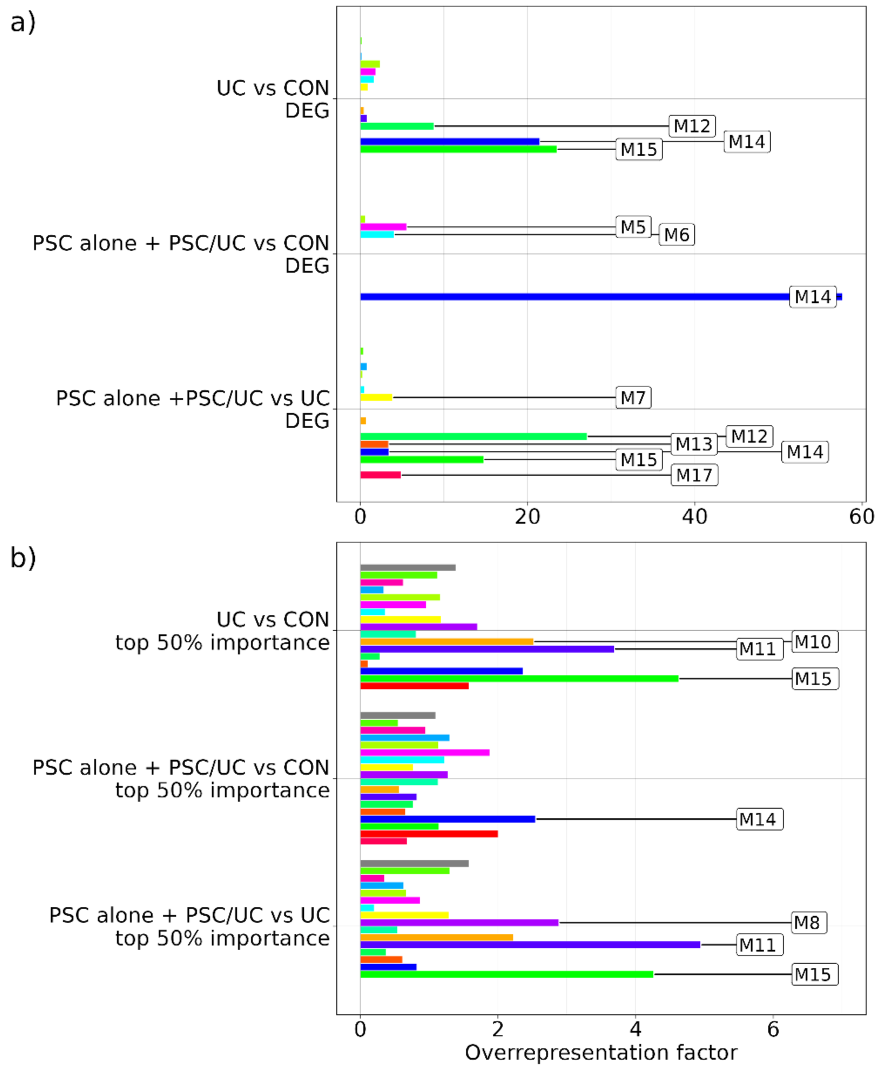

Fig. S5: Overrepresentation factors of differentially expressed genes (DEG) and random forest most important features highlight coexpression modules most relevant for distinction of UC, PSC and healthy controls. DEGs and top 50% cumulative feature importance (average Gini index impurity increase) gene sets of models (i) to (iii) were analysed. These modules represent transcriptional modules differentially activated in blood of PSC and UC patients and healthy controls (**Table 1**). Overrepresentation is defined as the ratio of observed genes in a module from a gene set divided by genes expected under a uniform distribution. If DEGs and most important random forest gene sets were independent of coexpression modules, each overrepresentation score would equal one on average.

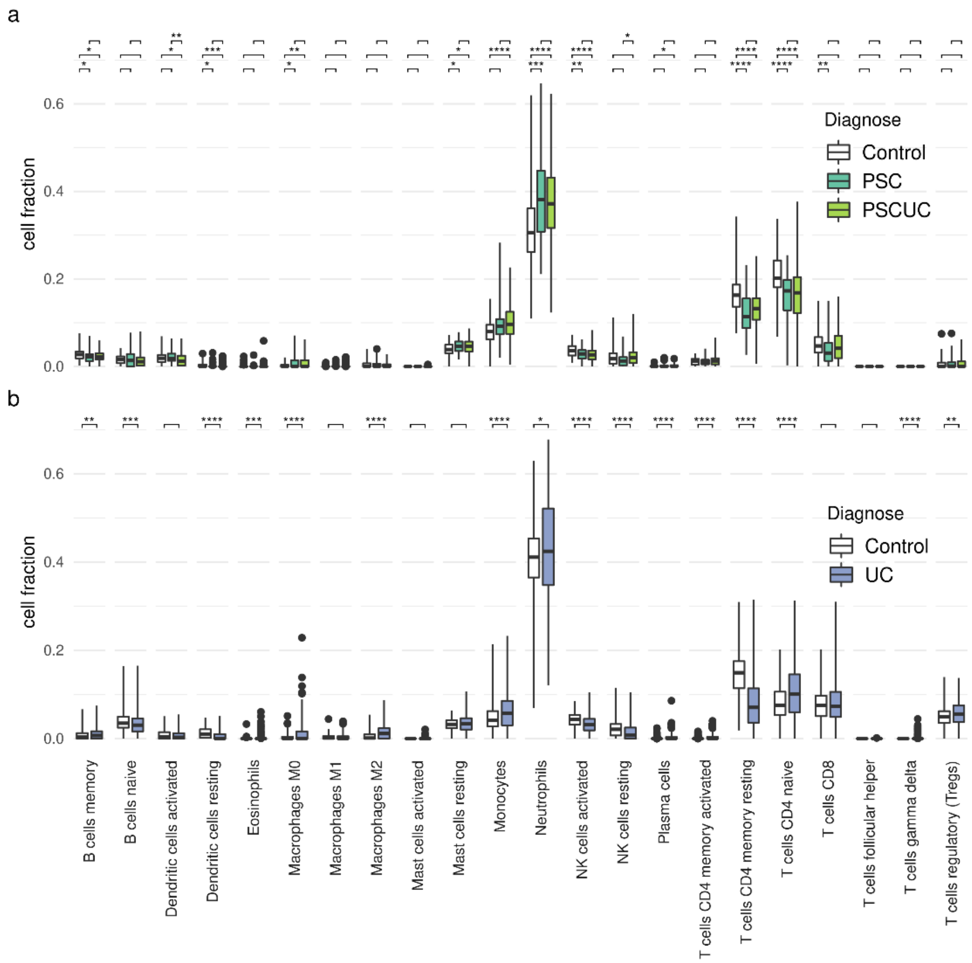

Fig. S6: Estimated cell fractions in the different Cohorts, grouped by Disease. Deconvolution with Cibersort[1] based on the LM22 signature matrix. Plot a) shows the estimates grouped by Diagnose for the PSC cohort. Plot b) shows the estimates grouped by Diagnose for the UC cohort. Direct comparison of PSC and UC samples is not possible, because log-scale z-scores, which we used for batch correction and analysis, are unsuitable as input for Cibersort.

Supplementary Methods

Downloading external data

We downloaded the expression tables of our validation studies using the R package GEOquery for the data hosted on GEO (GSE112057, GSE94648) and using the ftp-server ftp.sra.ebi.ac.uk/ to download the fastq-files from the European Nucleotide Archive (PRJEB28822). The fastq-files from ENA were processed into a count table using the same pipeline that was used for the main analysis, nf-core's rna-seq[2].

Cell Deconvolution (Cibersort)

Cibersort[1] was applied to estimate the relative fractions of cells of the samples. This supports the interpretation if a gene is upregulated due to cell abundance or differential expression in one or multiple cell types, or both. We ran Cibersort in group mode, using the LM22 matrix as provided on (https://cibersortx.stanford.edu/runcibersortx.php). Quantile normalization was disabled, as recommended for RNA-Seq data, and batch correction in B-mode was applied. Cell fractions of the patient cohorts were only compared to their respective controls because of batch effects, that could not be corrected for due to the Cibersort requiring input on a non-log, non-negative scale. Inputting the z-score centered-log-ratio values would lead to invalid results. Statistical comparison was performed with a Mann-Whitney U-test due to non-normality.

Cell-type specific enrichment (LM22)

We used the LM22 expression data to visualize if a gene is preferably expressed in a specific cell type. The LM22 expression data was used to calculate the cell deconvolution signature matrix in Newman *et al.* 2015[1]. It consists of 113 samples of 22 cell types of purified immune cells from PBMCs from peripheral blood of healthy donors. All samples were processed with the HGU133A microarray platform (Affymetrix). We normalized the gene expression matrix by gene, so the resulting z-score would indicate if a gene were more or less expressed than it is in other cell types. To summarize this information for gene sets like the Cemitool[3] coexpression modules, we calculated the arithmetic mean of the gene z-scores per cell type. We selected an absolute z-score threshold of 0.5 as significance threshold when assigning a module to a cell type.

Blood informative transcripts

Blood informative transcripts from Preininger *et al.*[4] were used as support for the biological interpretation of the coexpression modules. Briefly, Preininger *et al.* observed sets of genes that were coexpressed across transcriptomics datasets. They identified 10 representative transcripts (blood informative transcripts, BIT) for each of the 9 sets called “axes”. The transcripts were selected to not correlate with the other axes and to strongly correlate within the respective axis. We looked up in which of our coexpression modules these BITs are included and noted down any overlap of two or more genes in Supplementary Table S12.

Supplementary Results

Gut homing hypothesis

We looked up key genes implicated in the gut-homing hypothesis for expression in our dataset[5], Supplementary Table S20). CCR7 is downregulated in PSC vs controls and important in RF (rank 15). In UC, CX3CR1 and CXCR4 are downregulated, while IL17RA (M4) is upregulated. In blood, IL17RA is expressed in neutrophils and monocytes, and its activation appears to be stronger in UC overall.

Cell deconvolution suggests altered abundance of CD4 T cells in PSC and UC.

To understand the observed dysregulation in more detail, cell fractions were deconvoluted using Cibersort[1]. In whole blood bulk RNA-seq experiments, upregulation of a gene can be attributed to overexpression of the gene or a higher fraction of the cell types expressing it. In UC, a change in cell fractions is expected in blood[6]. Cell fractions were calculated using the LM22 cell signature matrix and the “B-mode” for batch correction. In this way, cell fractions were estimated for every sample. We observed that many cell types show significant changes in abundance, in both UC and PSC, irrespective of UC comorbidity. In UC, most cell types are estimated to be significantly different in fraction, but the effect size is usually moderate. As an exception, CD4 resting memory T cells are estimated to be far less abundant. Neutrophils are estimated to be only slightly expanded.

In PSC, monocytes are estimated to be more abundant, while CD4 T cells (resting memory and naïve) are fewer. CD4 activated memory T cells are unaffected. Neutrophils are predicted to be 22% (ratio of medians, 0.372/0.306) more abundant than in controls. This is significant, but not enough to explain log2fold-changes of 1.35 like we observed for *ANXA3*. In UC, neutrophils are less overabundant, while *ANXA3* and others show strong overexpression. We conclude that actual upregulation, not just cell fraction changes, are causing the differentially expressed genes with large effect sizes.

Supplementary References

Author names in bold designate shared co-first authorship.

1. Newman, A. M. *et al.* Robust enumeration of cell subsets from tissue expression profiles. *Nat. Methods* **12**, 453–457 (2015).
2. Ewels, P. *et al.* nf-core/rnaseq: nf-core/rnaseq version 1.3. (2019) doi:10.5281/ZENODO.2610144.
3. **Russo, P. S. T.** *et al.* CEMiTool: a Bioconductor package for performing comprehensive modular co-expression analyses. *BMC Bioinformatics* **19**, 56 (2018).
4. Preininger, M. *et al.* Blood-informative transcripts define nine common axes of peripheral blood gene expression. *PLoS Genet.* **9**, e1003362 (2013).
5. de Krijger, M., Wildenberg, M. E., de Jonge, W. J. & Ponsioen, C. Y. Return to sender: Lymphocyte trafficking mechanisms as contributors to primary sclerosing cholangitis. *J. Hepatol.* **71**, 603–615 (2019).
6. Hanai, H. *et al.* Relationship between fecal calprotectin, intestinal inflammation, and peripheral blood neutrophils in patients with active ulcerative colitis. *Dig. Dis. Sci.* **49**, 1438–1443 (2004).
