# Supplementary material for: Whole blood RNA sequencing identifies transcriptional differences between primary sclerosing cholangitis and ulcerative colitis
Source: JHEP Rep. 2023 Dec 19;6(2):100988. doi: 10.1016/j.jhepr.2023.100988 (PMC10832281; doi:10.1016/j.jhepr.2023.100988)
Supplement: Multimedia component 2 — : [file mmc2.docx]

**JHEP Reports**

**CTAT methods**

Tables for a “Complete, Transparent, Accurate and Timely account” (CTAT) are now mandatory for all revised submissions. The aim is to enhance the reproducibility of methods.

- Only include the parts relevant to your study
- Refer to the CTAT in the main text as ‘Supplementary CTAT Table’
- Do not add subheadings
- Add as many rows as needed to include all information
- Only include one item per row

**If the CTAT form is not relevant to your study, please outline the reasons why:**

|  |
| --- |

- 1. **Antibodies**

| **Name** | **Citation** | **Supplier** | **Cat no.** | **Clone no.** |
| --- | --- | --- | --- | --- |
| **---** |  |  |  |  |

- 1. **Cell lines**

| **Name** | **Citation** | **Supplier** | **Cat no.** | **Passage no.** | **Authentication test method** |
| --- | --- | --- | --- | --- | --- |
| **---** |  |  |  |  |  |

- 1. **Organisms**

| **Name** | **Citation** | **Supplier** | **Strain** | **Sex** | **Age** | **Overall n number** |
| --- | --- | --- | --- | --- | --- | --- |
| **---** |  |  |  |  |  |  |

- 1. **Sequence based reagents**

| **Name** | **Sequence** | **Supplier** |
| --- | --- | --- |
| **---** |  |  |

- 1. **Biological samples**

| **Description** | **Source** | **Identifier** |
| --- | --- | --- |
| **Human blood samples from Ulcerative colitis and primary sclerosing cholangitis patients, healthy controls** | **Germany, Norway** |  |

- 1. **Deposited data**

| **Name of repository** | **Identifier** | **Link** |
| --- | --- | --- |
| **Gene Expression Omnibus** | **GSE177044** | **https://www.ncbi.nlm.nih.gov/geo/query/acc.cgi?acc=GSE177044** |

- 1. **Software**

| **Software name** | **Manufacturer** | **Version** |
| --- | --- | --- |
| **Nf-core rna-seq pipeline** | **Nf-core** | **1.3** |
| **R** | **R core team** | **4.3.1** |
| **DESeq2** | **M. Love et al. Via BioConductor** | **1.38.3** |
| **CEMiTool** | **Russo et al. Via BioConductor** | **1.22.0** |
| **For version numbers of R packages please refer to https://github.com/ikmb/ucpsc-rnaseq/blob/71c2d58a29b2d54443c8a49860bbc664149423b2/01_R_session/UCPSC_main_script.r** |  |  |
|  |  |  |

- 1. **Other (*e.g*. drugs, proteins, vectors etc.)**

| **---** |  |  |
| --- | --- | --- |
|  |  |  |

- 1. **Please provide the details of the corresponding methods author for the manuscript:**

| David Ellinghaus, Prof. Dr. rer nat.  Institute of Clinical Molecular Biology (IKMB)  Kiel University (CAU) and University Medical Center Schleswig-Holstein (USKH)  Rosalind-Franklin-Str. 12, 24105 Kiel, Germany  E-mail: [d.ellinghaus@ikmb.uni-kiel.de](mailto:d.ellinghaus@ikmb.uni-kiel.de) |
| --- |

**2.0 Please confirm for randomised controlled trials all versions of the clinical protocol are included in the submission. These will be published online as supplementary information.**

| **---** |
| --- |
